# Post-Traumatic Growth and Post-Traumatic Stress Disorder in Acute Myocardial Infarction among Younger and Older Adults: A Retrospective Cohort Study

**DOI:** 10.62641/aep.v53i5.1859

**Published:** 2025-10-05

**Authors:** Kun Bai, Liang Wen

**Affiliations:** ^1^Hanzhong Central Hospital Coronary Care Unit (CCU), 723099 Hanzhong, Shaanxi, China

**Keywords:** post-traumatic growth, post-traumatic stress disorder, acute myocardial infarction, younger adult, older adult

## Abstract

**Background:**

While the physical implications of acute myocardial infarction (AMI) have been extensively studied, its psychological aspects, particularly post-traumatic growth (PTG) and post-traumatic stress disorder (PTSD) have gained increasing attention. This retrospective cohort study aimed to investigate the correlations between age, PTG, and PTSD in the context of AMI.

**Methods:**

A total of 250 cases of patients with AMI were included in the study, sourced from the coronary care unit of Hanzhong Central Hospital and followed up in the outpatient department from January 2017 to June 2023. The data collection for this study was conducted from July 2023 to August 2023. Patients were divided into two groups based on their age at the time of AMI: 148 patients in the Younger group (≤45 years) and 102 patients in the Older group (>45 years). The patients were assessed for PTSD using the PTSD Checklist-Civilian Version (PCL-C) and for PTG using the Posttraumatic Growth Inventory (PTGI). Statistical analysis was conducted to examine the correlations and associations between age and PTG and PTSD symptoms.

**Results:**

The findings revealed significant age-related variations in PTSD symptomatology and PTG following AMI. Older adults exhibited higher re-experience (*p* < 0.001), lower hyperarousal (*p* = 0.023), and lower avoidance/numbing (*p* = 0.037) symptoms compared to younger adults, along with decreased scores in PTG domains such as relating to others (*p* < 0.001), appreciation of life (*p* < 0.001), spiritual change (*p* < 0.001), and personal strength (*p* < 0.001). The correlation analysis further demonstrated that age was significantly positively correlated with re-experience (r = 0.366, *p* < 0.001) and negatively correlated with avoidance/numbing (r = –0.129, *p* = 0.041), hyperarousal (r = –0.154, *p* = 0.015), relating to others (r = –0.393, *p* < 0.001), appreciation of life (r = –0.256, *p* < 0.001), spiritual change (r = –0.285, *p* < 0.001), and personal strength (r = –0.460, *p* < 0.001). Linear regression analysis showed that for every year increase in age, the beta coefficient for re-experience was 0.369 (Standard Error (SE) = 0.051, *t* = 7.18, *p* < 0.001, 95% Confidence Interval (CI) [0.266, 0.466]), indicating a significant positive association. Conversely, age had significant negative associations with avoidance/numbing (β = –0.131, SE = 0.061, *t* = –2.11, *p* = 0.036, 95% CI [–0.249, –0.009]), hyperarousal (β = –0.158, SE = 0.067, *t* = –2.30, *p* = 0.022, 95% CI [–0.286, –0.022]), relating to others (β = –0.391, SE = 0.047, *t* = –8.36, *p* < 0.001, 95% CI [–0.485, –0.301]), appreciation of life (β = –0.263, SE = 0.058, *t* = –4.41, *p* < 0.001, 95% CI [–0.370, –0.142]), spiritual change (β = –0.282, SE = 0.054, *t* = –5.28, *p* < 0.001, 95% CI [–0.391, –0.179]), and personal strength (β = –0.464, SE = 0.049, *t* = –9.39, *p* < 0.001, 95% CI [–0.556, –0.364]).

**Conclusions:**

The study underscores the importance of adopting a multidimensional approach to patient care following AMI, tailored interventions to address the specific needs of younger and older adults, and the need for age-specific psychological assessment and intervention strategies in the management of patients recovering from AMI.

## Introduction

Acute myocardial infarction (AMI), commonly known as a heart attack, poses a significant global health threat and is a leading cause of mortality and morbidity across all age groups [1, 2]. The aftermath of AMI often extends beyond the physical implications, involving complex interactions between psychological and emotional responses in affected individuals [3]. While traditional research has primarily focused on identifying and managing the physical sequelae of AMI, the psychological aspects of the condition have garnered increasing attention in recent years [4, 5]. Specifically, the exploration of post-traumatic growth (PTG) and post-traumatic stress disorder (PTSD) has emerged as a vital area of study in understanding the holistic impact of AMI on patients-particularly age-related differences in psychological responses to this traumatic event [6, 7]. It is well established that there are age-related differences in the presentation of PTSD and PTG following AMI [8]. For instance, studies have reported that older MI patients experience hyperarousal most frequently, followed by avoidance and re-experiencing symptoms [9]. Furthermore, the findings demonstrate that older adults exhibit less pronounced positive psychological growth in domains such as positive affect and physical activity engagement, whereas midlife participants show greater improvements in these areas—consistent with the intervention’s outcomes of reduced depression and enhanced psychological well-being [10]. von Känel *et al*.’s [11] research showed that compared with AMI patients without acute stress disorder (ASD) or PTSD, those with these conditions are younger and have relatively milder coronary artery disease. These observations align with a growing body of evidence suggesting potential variations in the psychological responses to AMI among different age groups.

PTG represents the positive psychological change that individuals may experience following a traumatic event such as AMI [12, 13]. It encompasses various domains, including personal strength, relationships with others, appreciation of life, spiritual growth, and new possibilities, reflecting the multifaceted nature of growth that can emerge in the aftermath of trauma [14, 15]. Conversely, PTSD is characterized by a cluster of symptoms, including re-experiencing, avoidance/numbing, and hyperarousal, resulting from exposure to a traumatic event and often leading to significant distress and impairment in functioning [16, 17, 18]. While previous research has recognized the psychological impact of AMI, a comprehensive understanding of the interplay between PTG and PTSD in the specific context of AMI, particularly in relation to age-related differences, remains incompletely understood [19].

The differential impact of AMI on younger and older adults has been an area of growing interest, with emerging evidence suggesting potential variations in the psychological responses to this traumatic event [20, 21]. Younger adults (defined here as aged 45 and below) may face distinct challenges in coping with AMI compared to middle-aged and older counterparts, with potentially different manifestations of PTG and PTSD [22]. For example, studies have noted that the three pre-assessed psychological characteristics, namely neuroticism, sense of control, and self-efficacy expectations, have an impact on the functional decline of middle-aged and elderly patients with AMI after the onset of the diseases [23]. Therefore, investigating the nuances of PTG and PTSD in the context of age is imperative for tailoring comprehensive interventions that address the specific needs of patients within each age group. The mutual influence between age and psychological responses to AMI is of particular significance in light of the intricate interconnections between physical and psychological well-being. As described in the literature, AMI and its subsequent treatments can serve as a catalytic event for individuals to reflect on their lives, reevaluate their priorities, and potentially experience growth in unexpected ways. These processes may unfold differently across age groups, thereby necessitating age-specific approaches to psychological assessment and intervention for individuals recovering from AMI. This study aims to explore the correlations between age, PTG, and PTSD, clarifying these nuanced relationships in the context of AMI. Its insights may enrich the broader discourse surrounding the psychological implications of AMI and inform further research and clinical initiatives to promote comprehensive well-being in this population.

## Methods

### Study Design and Population

This retrospective analysis included 250 patients with AMI who were admitted to the coronary care unit of Hanzhong Central Hospital and regularly followed up in the outpatient department from January 2017 to June 2023. Data collection was conducted in two phases to ensure that all participants underwent psychological assessments at least six months post-AMI: Phase 1 (July 2023): We reviewed patient records to identify all eligible patients who had experienced AMI between January 2017 and December 2022. For these patients, PTSD Checklist-Civilian Version (PCL-C) and Posttraumatic Growth Inventory (PTGI) assessments were completed during their regular follow-up visits in July 2023, ensuring that at least six months had passed since their AMI event. Phase 2 (January 2024): For patients who experienced AMI from January 2023 to June 2023, we postponed data collection until January 2024 to ensure they also had at least six months post-AMI before undergoing psychological assessments.

The patients were divided into two groups based on their age at the time of AMI: the Young group (≤45 years, n = 148) and the Older group (>45 years, n = 102). The age range for the Young group was 18–45 years, while that for the Older group was 46–83 years.

The inclusion criteria were as follows: (1) Patients aged ≥18 years with AMI of Types 1, 2, 4b, or 4c (Type 4a myocardial infarction was excluded due to its iatrogenic nature, which may differ in psychological impact from spontaneous AMI [24]); (2) Haemoglobin levels <10 g/dL within 24 hours of the onset; (3) Survived myocardial infarction treatment and had normal or mildly depressed mental status; (4) Had at least a primary school level of education, normal cognitive function, and were able to cooperate and complete the study.

Exclusion criteria: (1) Required cardiopulmonary resuscitation on admission; (2) Were experiencing uncontrolled bleeding; (3) Were receiving palliative care; (4) Were planning for cardiac surgery during their hospitalization; (5) Were refusing blood transfusion; (6) Had a known history of PTSD or other mental illnesses; (7) Exhibited severe post-treatment confusion.

This phased approach ensured that all participants underwent evaluations at the appropriate time points, maintaining the integrity of the study design. This study was approved by the Ethics Committee of Hanzhong Central Hospital (Approval Number: [2024-126]). All procedures involving human participants were in accordance with the ethical standards of the institutional and/or national research committee and with the 2013 Helsinki Declaration, as well as its later amendments or comparable ethical standards. Informed consent was obtained from all individual participants included in the study.

### Routine Life Indicators

Demographic and socioeconomic indicators, including educational status, marital status, healthcare payment methods, residence, monthly family income, and occupation, were collected via a structured questionnaire. PTSD symptoms were independently assessed using the PCL-C according to its standardized scoring criteria. The PCL-C was developed in January 1994 based on the Diagnostic and Statistical Manual of Mental Disorders, Fourth Edition (DSM-IV) and is specifically designed to assess the experiences of trauma in everyday life (as opposed to during wartime) in the general population. It comprises 17 items categorized into three factors: re-experiencing includes 5 items (total score for this subscale ranges from 5 to 25), avoidance/numbing includes 7 items (total score for this subscale ranges from 7 to 35), and hyperarousal (total score for this subscale ranges from 5 to 25). The severity of each symptom is rated on a scale of 1 to 5, where 1 = not at all, 2 = a little bit, 3 = moderately, 4 = quite a bit, and 5 = extremely, as per the original PCL-C validation study [25]. The total scores for each subscale (re-experiencing: 5 items, 1–5 per item; avoidance/numbing: 7 items; hyperarousal: 5 items) were calculated by summing individual item scores, consistent with standard PCL-C scoring guidelines. Higher scores indicate a greater impact of stress on the individual’s psychological condition. The PCL-C assessment was conducted by well-trained clinical psychologists at the outpatient department of Hanzhong Central Hospital. The follow-up evaluation was conducted at least 6 months after the AMI event to ensure sufficient time had passed for psychological changes to manifest.

### Post-Traumatic Growth Indicators

In this study, we used the PTGI [26]. The PTGI comprises 21 items across five dimensions: personal growth (4 items), relating to others (7 items), spiritual change (2 items), new possibilities (5 items), and appreciation of life (3 items). Each item is scored on a 6-point Likert scale ranging from 0 (‘I did not experience this change at all’) to 5 (‘I experienced this change to a very great degree’), consistent with modified versions of the PTGI validated in previous studies [27]. The total score for each dimension is the sum of the scores of the items within that dimension. To standardize the scores, we converted the raw scores to a 100-point scale using the following formula: Standardized Score = (Raw Score/Maximum Possible Score) × 100. The PTGI inventory was used to assess patients’ PTG indicators, which include relating to others, appreciation of life, new possibilities, spiritual change, and personal strength. The PTGI assessment was conducted by well-trained clinical psychologists at the outpatient department of Hanzhong Central Hospital. The follow-up evaluation was conducted at least 6 months after the AMI event to ensure sufficient time had passed for psychological changes to manifest. Non-normally distributed PTGI scores were presented as median with interquartile range (IQR). Standardized scores were truncated to a maximum of 100 to align with the defined scale range (0–100).

### Statistical Methods

The data were analyzed using IBM SPSS Statistics Version 25.0 (IBM Corp., Armonk, NY, USA). Categorical data were presented as [n (%)] and assessed using the chi-square test with the basic formula when the sample size was ≥40 and the theoretical frequency T was ≥5, yielding a test statistic χ^2^. When the sample size was ≥40 but the theoretical frequency 1 ≤ T < 5, the chi-square test was conducted using the corrected formula. For sample sizes <40 or theoretical frequencies T <1, statistical analysis was performed using Fisher’s exact probability test. Normally distributed quantitative data was expressed as mean ± standard deviation (SD). For normally distributed data, *t*-tests were used for significance testing. For non-parametric data, the Wilcoxon rank-sum test was used, and the test statistic was denoted as W. For correlation analysis, Pearson correlation was used for continuous variables that conform to the normal distribution, while Spearman correlation was used for non-parametric data. Significant indicators showing statistical differences between the two groups were then subjected to linear regression analysis, with PTG and PTSD symptoms as dependent variables and age as the independent variable,respectively. Statistical significance was set at a *p* value of less than 0.05 for all analyses.

## Results

### Baseline Data and Demographic Characteristics

The baseline data and demographic characteristics of the patients in both groups are summarized in Table 1. No significant differences were observed between the Younger Group (n = 148) and the Older Group (n = 102) in gender distribution, smoking history, alcohol intake, hypertension, diabetes, education level, marital status, medical expense payment method, residence, family monthly income, and occupation. These findings indicate that baseline characteristics were well-balanced between the two groups, supporting the suitability of the study design for evaluating age-related psychological differences.

**Table 1.** **Baseline data and demographic characteristics of patients**.

Parameter		Younger group	Older group	*t*/χ^2^	*p* value
		(n = 148)	(n = 102)		
Age (years)		34.84 ± 5.17	62.94 ± 7.49	32.896	<0.001
Gender					
	Male	118 (79.73%)	83 (81.37%)	0.103	0.748
	Female	30 (20.27%)	19 (18.63%)		
Smoking history					
	Yes	52 (35.14%)	40 (39.22%)	0.432	0.511
	No	96 (64.86%)	62 (60.78%)		
Alcohol intake					
	Yes	85 (57.43%)	71 (69.61%)	3.815	0.051
	No	63 (42.57%)	31 (30.39%)		
Comorbidities (%)					
	Hypertension	29 (19.59%)	21 (20.59%)	0.037	0.847
	Diabetes	24 (16.22%)	20 (19.61%)	0.479	0.489
Education level					
	Junior high school or below	100 (67.57%)	73 (71.57%)	0.454	0.501
	Above junior high school	48 (32.43%)	29 (28.43%)		
Marital status					
	Married	138 (93.24%)	97 (95.10%)	0.492	0.782
	Divorced	5 (3.38%)	2 (1.96%)		
	Widowed	5 (3.38%)	3 (2.94%)		
Medical expense payment method					
	Out-of-pocket	4 (2.71%)	2 (1.96%)	0.000	>0.999
	Medical insurance	144 (97.29%)	100 (98.04%)		
Residence					
	Rural	68 (45.95%)	46 (45.10%)	0.018	0.895
	Urban	80 (54.05%)	56 (54.90%)		
Family monthly income					
	5000 CNY or below per month	9 (6.08%)	4 (3.92%)	0.571	0.450
	Above 5000 CNY per month	139 (93.92%)	98 (96.08%)		
Occupation					
	Worker	28 (18.92%)	17 (16.67%)	0.662	0.956
	Farmer	76 (51.35%)	54 (52.94%)		
	Clerk	14 (9.46%)	8 (7.84%)		
	Civil servant	5 (3.38%)	3 (2.94%)		
	Other	25 (16.89%)	20 (19.61%)		

1 CNY = 0.1374 USD.

### PCL-C Score

**Fig. 1.**
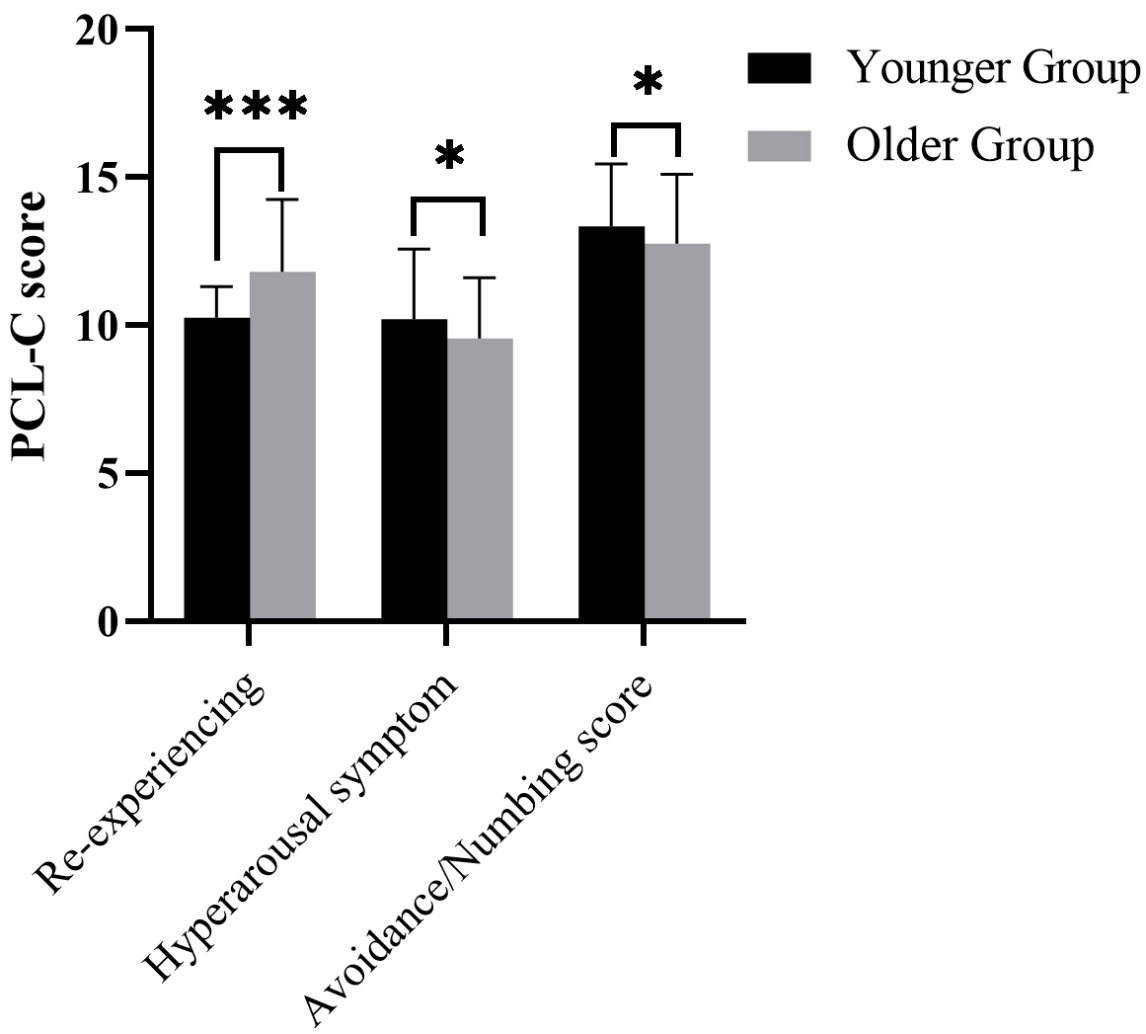
**Comparison of PCL-C score between the Younger group and Older group (PTSD), including three items of re-experience, hyperarousal symptoms and avoidance/numbing score**. PTSD, Post-traumatic stress disorder; PCL-C, PTSD Checklist-Civilian Version. *: *p* < 0.05 and ***: *p* < 0.001.

The comparison of PCL-C score between the Younger Group and the Older Group revealed significant differences in re-experience (*t* = 6.028; *p* < 0.001), hyperarousal (*t* = 2.281; *p* = 0.023), and Avoidance/Numbing (*t* = 2.093; *p* = 0.037) symptoms (Fig. 1). Specifically, the Older Group exhibits higher scores for re-experiencing (Younger Group: 10.26 ± 1.05; Older Group: 11.81 ± 2.45; range 5–25) and lower scores for hyperarousal (Younger Group: 10.21 ± 2.36; Older Group: 9.56 ± 2.04; range 5–25) compared to the Younger Group. The Avoidance/Numbing scores were significantly higher in the Younger Group (Younger Group: 13.34 ± 2.11; Older Group: 12.75 ± 2.36; range 7–35). These findings indicate age-related variations in PTSD symptoms, highlighting the importance of age-specific interventions for patients with PTSD.

### PTGI Scale

**Fig. 2.**
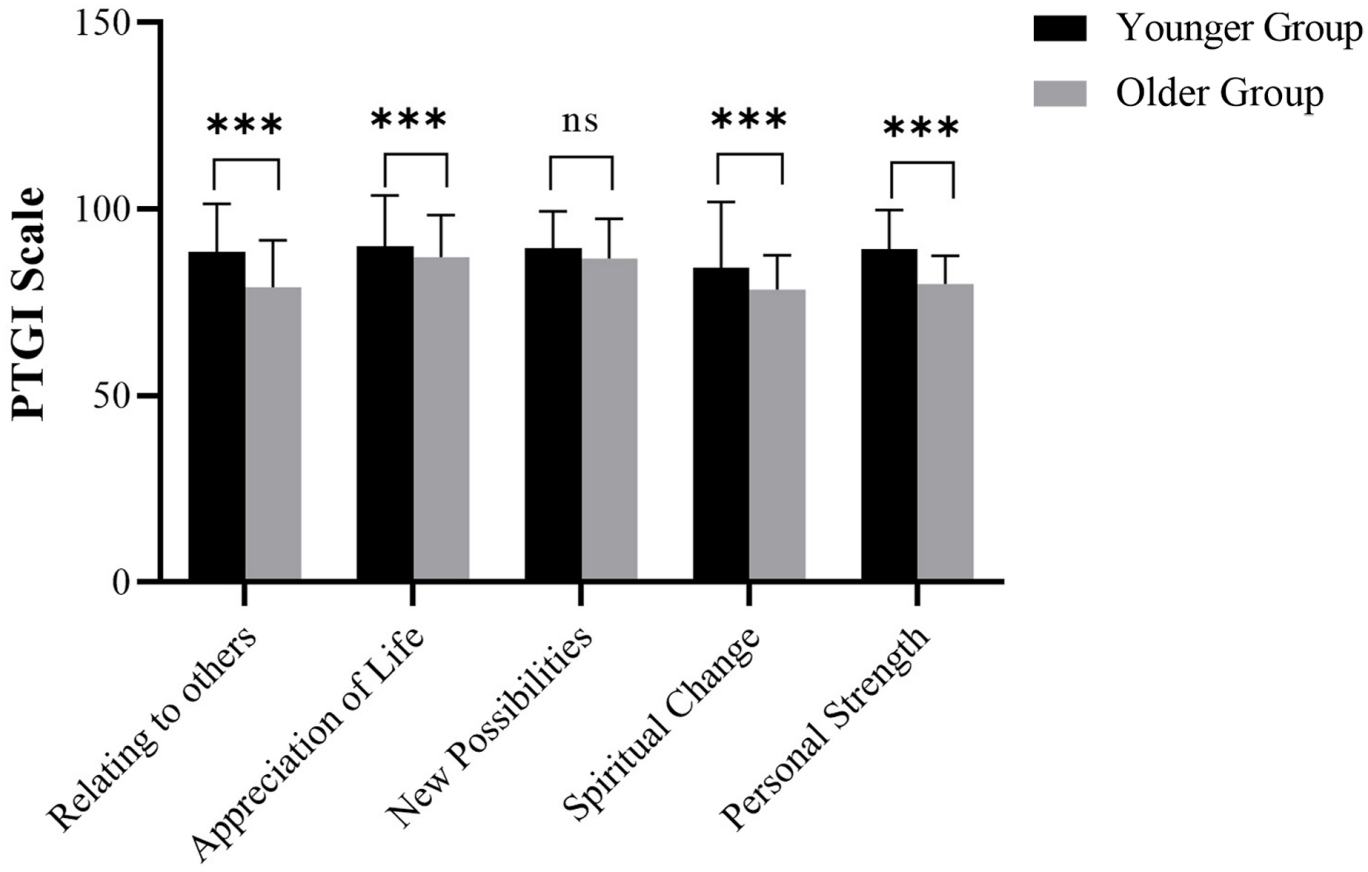
**Comparison of PTGI scale, including four items of relating to others, appreciation of life, new possibilities, spiritual change and personal strength**. PTGI, Posttraumatic Growth Inventory. ns, no significant difference and ***: *p* < 0.001.

The comparison of the PTGI Scale between the Younger Group and the Older Group revealed statistically significant differences in most domains (Fig. 2). Specifically, relating to others (Younger Group: 92.88 (80.00, 100.00); Older Group: 79.94 (72.34, 87.11); W = 10,842; *p* < 0.001), appreciation of life (Younger Group: 97.59 (81.78, 100.40); Older Group: 89.35 (80.52, 95.11); W = 9860.5; *p* < 0.001), spiritual change (Younger Group: 88.35 (73.11, 94.27); Older Group: 79.02 (73.15, 85.43); W = 10,069; *p* < 0.001), and personal strength (Younger Group: 91.78 (82.06, 99.70); Older Group: 80.05 (75.01, 85.03); W = 11,615.5; *p* < 0.001) scores were significantly lower in the Older Group compared to the Younger Group. In contrast, there was no significant difference in the ‘New Possibilities’ domain scores between the two groups (Younger Group: 91.26 (82.95, 99.46); Older Group: 88.39 (80.75, 95.62); W = 8436.5; *p* = 0.113). These findings suggest age-related variations in posttraumatic growth following trauma, emphasizing the need for age-tailored interventions to promote posttraumatic growth in patients across different age groups.

### Correlational Analysis

Correlation analysis revealed significant associations between age and several PTSD parameters in patients with AMI (Table 2). Specifically, age correlated positively with re-experiencing symptoms (r = 0.366, *p* < 0.001), age correlated negatively with avoidance/numbing (r = –0.129, *p* = 0.041) and hyperarousal (r = –0.154, *p* = 0.015), appreciation of life (r = –0.256, *p* < 0.001) and personal strength (r = –0.460, *p* < 0.001) exhibited significant negative correlations with age, new possibilities (r = –0.100, *p* = 0.113) did not show a significant correlation with age, relating to others (r = –0.393, *p* < 0.001) and spiritual change (r = –0.285, *p* < 0.001) also showed significant negative correlations with higher age.

**Table 2.** **Correlation analysis of higher age and post-traumatic growth parameters and post-traumatic stress disorder parameters in patients with acute myocardial infarction**.

Parameter	Spearman’s rank correlation coefficient (rho)	*p* value
Re-experiencing	0.366	<0.001
Avoidance/Numbing	–0.129	0.041
Hyperarousal	–0.154	0.015
Relating to others	–0.393	<0.001
Appreciation of life	–0.256	<0.001
New possibilities	–0.100	0.113
Spiritual change	–0.285	<0.001
Personal strength	–0.460	<0.001

### Linear Regression Analysis

The linear regression analysis conducted to assess the relationship between age and PTSD parameters in patients with AMI revealed several significant findings. Age was significantly positively correlated with re-experience symptoms (β = 0.369, Standard Error (SE) = 0.051, *t* = 7.18, *p* < 0.001, 95% Confidence Interval (CI) [0.266, 0.466]), suggesting older AMI patients may experience more frequent or intense re-experiences of their trauma. Additionally, there were significant negative correlations between age and the following PTSD parameters: avoidance/numbing (β = –0.131, SE = 0.061, *t* = –2.11, *p* = 0.036, 95% CI [–0.249, –0.009]), hyperarousal (β = –0.158, SE = 0.067, *t* = –2.30, *p* = 0.022, 95% CI [–0.286, –0.022]), relating to others (β = –0.391, SE = 0.047, *t* = –8.36, *p* < 0.001, 95% CI [–0.485, –0.301]), appreciation of life (β = –0.263, SE = 0.058, *t* = –4.41, *p* < 0.001, 95% CI [–0.370, –0.142]), spiritual change (β = –0.282, SE = 0.054, *t* = –5.28, *p* < 0.001, 95% CI [–0.391, –0.179]), and personal strength (β = –0.464, SE = 0.049, *t* = –9.39, *p* < 0.001, 95% CI [–0.556, –0.364]). These findings suggest that as age increases, the likelihood of experiencing these PTSD-related symptoms (avoidance/numbing and hyperarousal) decreases.

Notably, new possibilities (β = –0.105, SE = 0.063, *t* = –1.59, *p* = 0.113, 95% CI [–0.224, 0.024]) did not show a statistically significant relationship with age, indicating no clear association between age and the perception of new opportunities following AMI (Table 3).

**Table 3.** **Linear regression analysis of age and post-traumatic stress disorder parameters in patients with acute myocardial infarction**.

Parameter	Beta (β)	Standard Error (SE)	*t*-value	*p* value	95% Confidence Interval (CI) for β
Re-experiencing	0.369	0.051	7.18	<0.001	[0.266, 0.466]
Avoidance/Numbing	–0.131	0.061	–2.11	0.036	[–0.249, –0.009]
Hyperarousal	–0.158	0.067	–2.30	0.022	[–0.286, –0.022]
Relating to others	–0.391	0.047	–8.36	<0.001	[–0.485, –0.301]
Appreciation of life	–0.263	0.058	–4.41	<0.001	[–0.370, –0.142]
New possibilities	–0.105	0.063	–1.59	0.113	[–0.224, 0.024]
Spiritual change	–0.282	0.054	–5.28	<0.001	[–0.391, –0.179]
Personal strength	–0.464	0.049	–9.39	<0.001	[–0.556, –0.364]

## Discussion

The present study sought to shed light on the nuanced relationships between age, PTG, and PTSD in the context of AMI. By delineating the psychological responses to AMI among younger and older adults, our findings underscore the intricate interplay between age-related factors and psychological sequelae following this traumatic event. Analysis of baseline and demographic characteristics revealed well-balanced distributions between the two groups, supporting the validity of comparing age-related psychological differences. The study indicated that tailored interventions may be necessary to address distinct symptoms and outcomes among younger and older adults recovering from AMI.

In the current study, we found significant differences between younger and older adults in terms of PTG and PTSD symptoms. These differences may be attributed to a variety of factors, including psychological maturity, life experiences, coping mechanisms, and social support. Older adults, with greater life experience, may have more refined coping strategies for dealing with trauma, which could explain the higher re-experience scores but lower hyperarousal scores compared to younger adults. Younger patients, on the other hand, may struggle more with avoidance/numbing, possibly due to less established coping skills or insufficient social support [28].

Interestingly, the comparison of PCL-C score between the Younger group and the Older group unveiled significant differences in re-experience, avoidance/numbing, and hyperarousal symptoms, indicating age-related variations in PTSD symptomatology. Notably, the Older group exhibited higher re-experience scores and lower hyperarousal scores compared to the Younger group, along with increased avoidance/numbing symptoms in the Younger group. The findings are consistent with existing literature of Jacquet-Smailovic M *et al*. [29] and highlight the differing PTSD symptoms resulting from AMI across different age groups. This may be attributed to the greater psychological maturity of older patients. Notably, the Older Group exhibited lower scores in relating to others, appreciation of life, spiritual change, and personal strength, as well as higher scores for re-experience and lower scores for hyperarousal compared to the Younger Group. These differences may stem from older adults’ greater psychological maturity, as suggested by Mahmud I *et al*. [30], highlighting the need for age-tailored psychological assessments and interventions.

The significant positive correlation between age and re-experience symptoms, alongside negative correlations between age and avoidance/numbing and hyperarousal symptoms, indicates that age influences the manifestation of PTSD symptoms following AMI. Additionally, the negative associations between PTG domains and age suggest that older age diminishes the impact of PTGI domains in AMI patients. Correlation and regression analyses revealed that age positively correlates with re-experiencing symptoms but negatively correlates with avoidance/numbing, hyperarousal, and most PTG domains (e.g., relating to others, personal strength). These associations indicate that age shapes both PTSD and PTG manifestations post-AMI. Together, these findings underscore the intricate interplay between age, PTG, and PTSD, emphasizing the need to integrate these factors into AMI patient management.

Clinically, the findings suggest that a one-size-fits-all approach to post-AMI care is inadequate. Instead, healthcare providers should adopt a personalized strategy, taking into account the unique psychological needs of different age groups [31, 32]. For older adults, interventions could focus on enhancing social connections by encouraging participation in community activities and support groups to foster a sense of belonging and reduce feelings of isolation [33]. Additionally, providing access to chaplaincy services or spiritual counseling can help individuals find meaning and purpose following an AMI. Implementing programs that foster gratitude and positive thinking, such as journaling or gratitude practices, may also enhance life satisfaction and personal growth. For younger adults, targeted interventions might include CBT to address avoidance/numbing behaviors and assist patients in confronting and processing their traumatic experiences. Psychoeducation about the nature of AMI and common psychological reactions to such events can reduce anxiety and fear. Teaching stress management techniques, such as deep breathing, progressive muscle relaxation, and mindfulness, can help manage hyperarousal and stress. By tailoring interventions to the specific needs of each age group, clinicians can better support the psychological recovery of AMI patients, leading to improved overall well-being and quality of life.

The implications of these findings for clinical practice are multifaceted. First, adopting a multidimensional approach to patient care following AMI involves considering both physical and psychological aspects. Healthcare providers can implement age-specific assessments to identify unique needs and develop tailored interventions for younger and older adults. For example, younger patients might benefit from interventions focusing on building resilience and coping mechanisms, whereas older patients might require support in managing re-experience symptoms and fostering social connections. Second, by integrating these targeted approaches, healthcare providers can better promote psychological resilience and mitigate distress in patients following AMI, thereby improving overall well-being. These implications align with the evolving paradigm of patient-centered care, wherein holistic well-being, including psychological and emotional aspects, is integral to the management of cardiovascular diseases such as AMI. Moreover, the study’s findings have significant implications for the broader discourse surrounding the psychological implications of AMI. By illuminating the interplay between age and PTG and PTSD in the context of this traumatic event, the study contributes to a more comprehensive understanding of the holistic impact of AMI on patients. Furthermore, the study enhances the existing literature by demonstrating age-related variations in PTG and PTSD following AMI, contributing to a deeper understanding of the psychological responses to this critical health event. Future studies could explore additional factors influencing PTG and PTSD in the context of AMI, such as social support, coping strategies, and pre-existing psychological resilience, to further refine our understanding of the psychological responses to AMI across different age groups.

However, the present study provides valuable insights into the relationship between age, PTG, and PTSD in the context of AMI, several limitations must be acknowledged. First, the study’s retrospective design limits our ability to establish causal relationships between age and psychological outcomes. Prospective studies with longer follow-up periods would be beneficial to further investigate these associations. Second, the sample size, though adequate for statistical comparisons, may not fully represent the diversity of AMI patients across different regions and cultures. Third, the use of self-report measures for assessing PTG and PTSD introduces the possibility of bias due to subjective reporting. Future studies incorporating objective measures and clinical interviews could provide more robust data. Fourth, a significant limitation is the lack of information regarding the duration of the rehabilitation period for the enrolled patients. The specific timeframe during which participants were involved in rehabilitation after their AMI was not specified, which is crucial for understanding how rehabilitation might influence the observed psychological outcomes. Including detailed records of the rehabilitation period in future research would help to better assess the impact of rehabilitation on PTG and PTSD in different age groups. Lastly, the study did not control for the impact of social support and coping strategies, which are known to influence psychological outcomes. Including these variables in future research could provide a more comprehensive understanding of the factors affecting PTG and PTSD in AMI patients.

From a research perspective, future studies could explore additional factors influencing PTG and PTSD in the context of AMI, such as social support, coping strategies, and pre-existing psychological resilience, to further refine our understanding of the psychological responses to AMI across different age groups. Additionally, longitudinal studies could provide insights into the long-term trajectories of PTG and PTSD following AMI across different age cohorts, elucidating the enduring impacts of this traumatic event on patients’ psychological well-being. By deepening our understanding of the psychological responses to AMI, future research endeavors can contribute to the development of targeted interventions that address the complex interplay of PTG and PTSD, thereby enhancing the comprehensive care and support provided to individuals affected by this critical health event.

## Conclusions

In conclusion, the results of this retrospective cohort study provide a nuanced understanding of the interconnections between age, PTG, and PTSD in the context of AMI, elucidating age-related differences in the psychological responses to this critical health event. By recognizing the complex interplay between age, PTG, and PTSD, healthcare professionals can tailor interventions to address the specific psychological and emotional needs of younger and older adults recovering from AMI, thereby promoting comprehensive well-being in this population. Furthermore, the findings from this study have the potential to enrich clinical initiatives aimed at promoting overall well-being in patients recovering from AMI, as well as paving the way for further research efforts in this domain.

## Availability of Data and Materials

The datasets used and/or analyzed during the current study are available from the corresponding author on reasonable request.
